# The inhibitory effects of *Staphylococcus aureus* on the antibiotic susceptibility and virulence factors of *Pseudomonas aeruginosa*: A549 cell line model

**DOI:** 10.1186/s13568-021-01210-y

**Published:** 2021-03-30

**Authors:** Sanaz Dehbashi, Mohammad Yousef Alikhani, Hamed Tahmasebi, Mohammad Reza Arabestani

**Affiliations:** 1 Department of Microbiology, Faculty of Medicine, Hamadan University of Medical Sciences, Fahmideh avenue, Hamadan, Iran; 2 School of Medicine, Shahroud University of Medical Sciences, Shahroud, Iran

**Keywords:** *Staphylococcus aureus*, *Pseudomonas aeruginosa*, Polymicrobial infections, Respiratory Tract, Resistance, Virulence

## Abstract

*Pseudomonas aeruginosa* and *Staphylococcus aureus* often lead to serious lung infections. This study aimed to investigate the role of *S. aureus* in the expression of the β-lactamase enzymes and virulence factors of *P. aeruginosa* in the polymicrobial infections of the respiratory tract. Biofilm and planktonic co-culture of *P. aeruginosa* and *S. aureus* were performed in the A549 cell line. Then, antibiotic resistance and virulence factors of *P. aeruginosa* were examined, and the expression of *lasR*, *lasI, algD*, *mexR,* and *KPC* genes were determined using qPCR. *S.aureus* decreased β-lactam resistance but increased resistance to tobramycin in the biofilm condition. Furthermore, *S.aureus* showed a positive effect on reducing resistance to meropenem, doripenem, and tobramycin (except PA-2). Altough it was demonstrated that *S.aureus* reduced the viability of *P. aeruginosa*, particularly in the biofilm state, the pathogenicity of the recovered strains of *P.aeruginosa* increased. Moreover, the gene expression levels for *lasR/I* and *algD* were increased in biofilm conditions. The levels of *lasI* were more prominent in the virulent strain than the β-lactamase producing strain. Furthermore, the expression of *KPC* was increased in all strains of *P. aeruginosa*. According to the findings of this study, *S. aureus* has an inhibitory effect in polymicrobial infections by suppressing the β-lactamase genes and viability of *P. aeruginosa*. Also, it cooperates with the biofilm-producing *P. aeruginosa* strains to increase pathogenicity and resistance to tobramycin.

## Introduction

The interaction among microorganisms involved in an infection, a polymicrobial infection, could worsen the disease's outcomes. The polymicrobial infection, including respiratory tract, wounds, and diabetic foot, consists of different microorganisms, which the most common ones are *Pseudomonas aeruginosa* and *Staphylococcus aureus* (Steindler et al. 2009; Nickol et al. 2019; Tahmasebi et al. 2020a, b, c).

Regarding the nature of the polymicrobial infections, biofilm formation is a typical characteristic. The interaction between *S. aureus* and *P.aeruginosa* induces the pathways, which initiate biofilm formation (Yang et al. 2011; Dehbashi et al. 2020). Evidence shows that their virulence increases, and antibiotic treatments become less efficient during co-infections (Koley et al. 2011; Armbruster et al. 2016; Dehbashi et al. 2020).

*P. aeruginosa* inhibits the electron chain transport of respiration using 4-hydroxy-2-heptylquinoline-N-oxide, kills *S. aureus* to acquire iron using the LasA protease, and produces rhamnolipids to disperse the *S. aureus* biofilm (Tognon et al. 2019; Woods et al. 2019). *S. aureus*, on the other hand, increases its resistance to the inhibition of respiration by forming small colony variants and ferments carbon sources to lactic acid as the end product (Frapwell et al. 2018; Tahmasebi et al. 2020a, b, c). *P. aeruginosa* establishes a biofilm within the lung of the susceptible patient coupled with the overproduction of the exopolysaccharide alginate (Ali Mirani et al. 2018; Tahmasebi et al. 2019). Moreover, the pathogenicity and antibiotic resistance of *P. aeruginosa* increases in polymicrobial infections (Hotterbeekx et al. 2017).

Antibiotic resistance and pathogenicity are influenced during the infection. To illustrate, the overproduction of the exopolysaccharide alginate from *P. aeruginosa* in the dual lung infections changes the susceptibility of *S. aureus* to vancomycin (Tognon et al. 2019; Woods et al. 2019). The Resistance-Nodulation-Division (RND) type efflux pumps are down-regulated during the co-culture. Also, the co-nfection of *S.aureus* and *P.aeruginosa* leads to antibiotic resistance and wound repair delay (Tognon et al. 2019). Various studies have demonstrated that the dual infection of *P. aeruginosa* and *S. aureus* is more virulent and resistant than those of single species (Ali Mirani et al. 2018; Tahmasebi et al. 2019). For example, some virulence factors of *P. aeruginosa,* including LasA protease, play a more beneficial role in enhancing beta-lactamase resistance (Alves et al. 2018). However, more data are needed to conclude the alteration of antibiotic resistance in the biofilm (Hotterbeekx et al. 2017).

Therefore, this study aims to investigate the resistance to carbapenems and tobramycin and the pathogenicity of *P. aeruginosa* co-cultured with *S. aureus* in a cell-culture model.

## Materials and methods

### Preparation of the standard strains

Some standard strains including *P. aeruginosa* PAO1, *S. aureus* ATCC43432, *P. aeruginosa* NCTC13359 (a strong biofilm-former and KPC^1^-producing strain) (PA-2), *P. aeruginosa* NCTC13618 (a toxin and KPC-producing strain) (PA-3), and *P. aeruginosa* NCTC 12,903 (a KPC-producing strain) (PA-4) were used in this study. All the strains were derived from clinical isolates and incubated at 37 °C and 200 rpm unless described otherwise. Trypticase Soy Broth (supplemented by 1% agar when needed) was applied to culture the bacterial strains. Mannitol salt agar (MSA) and cetrimide agar (CA) were used to recover *S. aureus* and *P. aeruginosa*, respectively. This study was approved by the Ethics Committee of Hamadan University of Medical Sciences (No: IRUMSHA. REC. 1396.694).

### A549 cell culture

The A549 cell line was obtained from the Pasteur Institute of Iran. It was cultured on cell culture treated flasks (Biofil, Korea) in the RPMI1640 (DNA BioTech, Iran) supplemented with 5% FBS (Invitrogen, USA) and penicillin–streptomycin (to a final concentration of 50–100 IU/mL for the former one and 50–100 µg/mL for the last one) (Sigma, USA). As the cells reached 90% confluence, they were treated by trypsin–EDTA (0.25%) (Sigma, USA). The cells were centrifuged at 1000*g* for 5 min; then the pellet was resuspended in RPMI1640 supplemented with 5% FBS. The cell suspension then was aliquoted in 24 wells cell culture plate and incubated at 37 °C and 5% CO_2_ until the cell monolayer formed.

### Co-culture of *P. aeruginosa* and *S. aureus* on the A549 cell line

The co-culture assays were done as described by the study of Anderson et al. (Anderson et al. 2008). Briefly, the bacterial strains were cultured on a TSB medium overnight and then centrifuged at 10000 g for five minutes. The pellets were suspended in a minimal essential medium (MEM) supplemented by l-glutamine and the OD_600_ was adjusted to 0.1. 250 µl of each bacterial suspension was added to the A549 monolayer and incubated at 37 °C and 5% CO_2_. In one- and five-hour time intervals after incubation, the supernatants were removed and replaced by fresh MEM + L-Glu. The plates were incubated for 24 h, and then the supernatants were collected, serially diluted in PBS, and plated on MSA and CA for eighteen hours to count the recovered colonies. The established biofilms were treated with 0.1% Triton X-100 in PBS and shaken vigorously for thirty minutes, then diluted and plated as described for the planktonic state. All the tests were done in triplicate.

### Biofilm assay of the recovered *P. aeruginosa* strains

A 500-µl sample of an overnight stationary phase broth culture was diluted 1:100 in a fresh and sterile broth media, which was then grown to mid-exponential phase (OD_600_ 0.7–1.0) at 37 ºC. 200 µl of this culture was pipetted into each well of a 96-well microtiter plate, then incubated for four hours at 37 ºC. After incubation, the contents of the wells were gently aspirated. Each well was washed three times with 200 µl of sterile phosphate-buffered saline. 200 µl of safranin-O dye was then pipetted into each well to stain any resultant biofilm, and the wells were then rinsed out with tap water. The plate was then dried in an incubator. Next, 200 µl 70% ethanol was added and the plate was shaked (100 rpm, 15 min). The resultant solution in the microtiter plate wells was then read using a plate reader, and the results were recorded (Yang et al. 2011). All tests were done in triplicate.

### Virulence factor production assay of the recovered *P. aeruginosa* strains

The productions of pyocyanin, pyoverdine, biofilm, LasA, and LasB were examined for the recovered strains described in the study of Dehbashi et.al (Dehbashi et al. 2020). Briefly, to investigate LasB production, 1% skim milk was added to the BHI agar, and the isolates were cultured and incubated at 37 °C for 18 h. The clear zone surrounded the colonies showed the positive test. The staphylolytic activity of LasA was detected by a spectrophotometry method. The overnight broth culture of *Staphylococcus aureus* ATCC25923 was adjusted to OD_600_:0.8. Then, the cultures of *P. aeruginosa* strains were suspended in a solution (pH: 7.4) containing glucose (30 mM), NaCl (8 mM), K2HPO4(60 mM),KH2PO4(35 mM), ZnCl2(0.025 mM), (NH4)2SO2(15 mM), l-glutamine (7 mM), CaCl2(0.05 mM), FeCl3(0.017 mM), C6H5Na3O7(35 mM), MgCl2(1.4 mM), thiamine (0.15 mM), dl-arginine (0.22 mM), uracil (0.2 mM), and nicotinic acid (0.1 mM). This solution stimulates LasA Staphylolytic activity. Then, 100 µl of this solution was added to 900 µl of Staphylococcal suspension, and the decrease in absorbance of the solution was monitored at OD595.

Pyocyanin concentration (µg/mL) was measured using the chloroform/HCL method. The OD_520_ of extracted samples were multiplied to 17.072 (Dehbashi et al. 2020). Also, to measure the pyoverdine level of *P. aeruginosa* strains, the recovered bacteria were inoculated to the RPMI1640 (Invitrogen, USA) and incubated at 37 °C and 100 rpm overnight. Then, the OD_600nm_ of the broth cultures were quantified. The cultures were centrifuged at 200 g for 30 min, and after filtering (0.22 µm Millipore filters, Merck, Germany), the OD_405_ was measured. The Relative Pyoverdine Production (RPP) was calculated using RPP: OD_405_/OD_600_ (Dehbashi et al. 2020). All tests were done in triplicate.

The alginate production was measured based on the study of valentine et al. Briefly, *P.aeruginosa* strains were cultured in Pseudomonas Isolation Broth (PIB) for 72 h. Three volume of ethanol was used to precipitate the Alginic acid. Then, the precipitant was filtered and dried in a vacuum oven. The dried weight of alginate was measured as µg/mL.

### Determination of the minimal inhibitory concentration of the recovered *P. aeruginosa* strains

Antibiotic disks (MAST, UK) and E-tests (Liofilchem, Italy) were used to examine the strains` susceptibility to several antibiotics before and after their co-culture growth. The recovered bacteria from planktonic and biofilm conditions of co-culture were employed for antibiotic susceptibility testing using disk diffusion and MIC based on CLSI 2018. Antibiotic susceptibility was performed for imipenem, meropenem, doripenem, and tobramycin. *P. aeruginosa* ATCC 27853 was used as the reference strain. All tests were done in triplicate.

### RNA extraction and gene expression of the recovered *P. aeruginosa* strains

The total RNA was isolated during the log phase of the mono-cultures and the co-cultures. The strains were inoculated into LB broth (Merck, Germany) and then incubated at 37 °C. The RNA was extracted, and cDNA synthesis was performed using the GeneAll RNA extraction kit and the GeneAll cDNA synthesis kit (GeneAll, Korea) according to the manufacturer's instructions. Quantitative real-time PCR was used to determine the expressions of *lasR*, *lasI, algD*, *mexR, and KPC* genes using the SYBR Green method, and *rpoD* was employed as the reference gene. The primers used from different studies (Savli et al. 2003; Bustin et al. 2005; Steindler et al. 2009; Choudhury et al. 2016; Falahat et al. 2016; Dehbashi et al. 2020) and are listed in Table 1. Each reaction contained 3 µL molecular grade water, 2 µL primers with a final concentration of 0.5 µM, and 10 µL SYBR Green master mix (Takara Bio, Inc., Otsu, Japan). The ABI Step One-Plus Light Cycler 96 (Applied Biosystems, Foster City, USA) was used. The cycling parameters included one denaturing cycle at 94 °C for 15 minutes, followed by 40 three-step amplification cycles (95 °C/30 s, 59 °C/30 s, and 72 °C/30 s). A melting curve was also drawn on the first run for each sample. The melting curve analysis was performed using a temperature range of 65 °C to 90 °C at a ramp rate of 0.3 °C/s. *P. aeruginosa* ATCC 27,853 was used as the negative control. All tests were done in triplicate.

**Table 1:** List of primers used in this study

Gene	Sequence	References
*lasI*	F: GAAATCGATGGTTATGACGC R: CGGCACGGATCATCATCTTC	Steindler et al. 2009
*lasR*	F: AAGTGGAAAATTGGAGTGGAG R: GTAGTTGCCGACGACGATGAAG	Dehbashi 2020
*mexR*	F: TCAGAACCTGAAACAAGGTTG R: ATCGCCGGCGTTTTTCATTGTG	Oldak 2005
*KPC*	F: CGTCTAGTTCTGCTGTCTTG R: TTGTCATCCTTGTTAGGCG	Tahmasebi et al. 2020a, b, c
*algD*	F: T GTCGCGCTACTACATGCGTC R: GTGTCGTGGCTGGTGATGAGA	Savli et al. 2003
*rpoD*	F: GGGCGAAGAAGGAAATGGTC R: CAGGTGGCGTAGGTGGAGAA	Wen 2018

### Statistical analysis

All the data were presented as mean ± SEM. For all the data collected, a two-way analysis of variance (ANOVA) was performed using GraphPad Prism 6.0 (Graph Pad Software, USA). When necessary, Tukey's test, the chi-square test, and the Student's t-test were applied to the data to determine the statistically significant changes by providing the adjusted P-values ($\le 0.01)$. All the presented P-values were adjusted for multiple comparisons. Gene expression analysis was performed using the REST® software (version 2009, Qiagen, Germany). The 2^−ΔΔCt^ method was used to determine the expression levels.

## Results

### Long-term competition on the A549 cell line

**Fig. 1:**
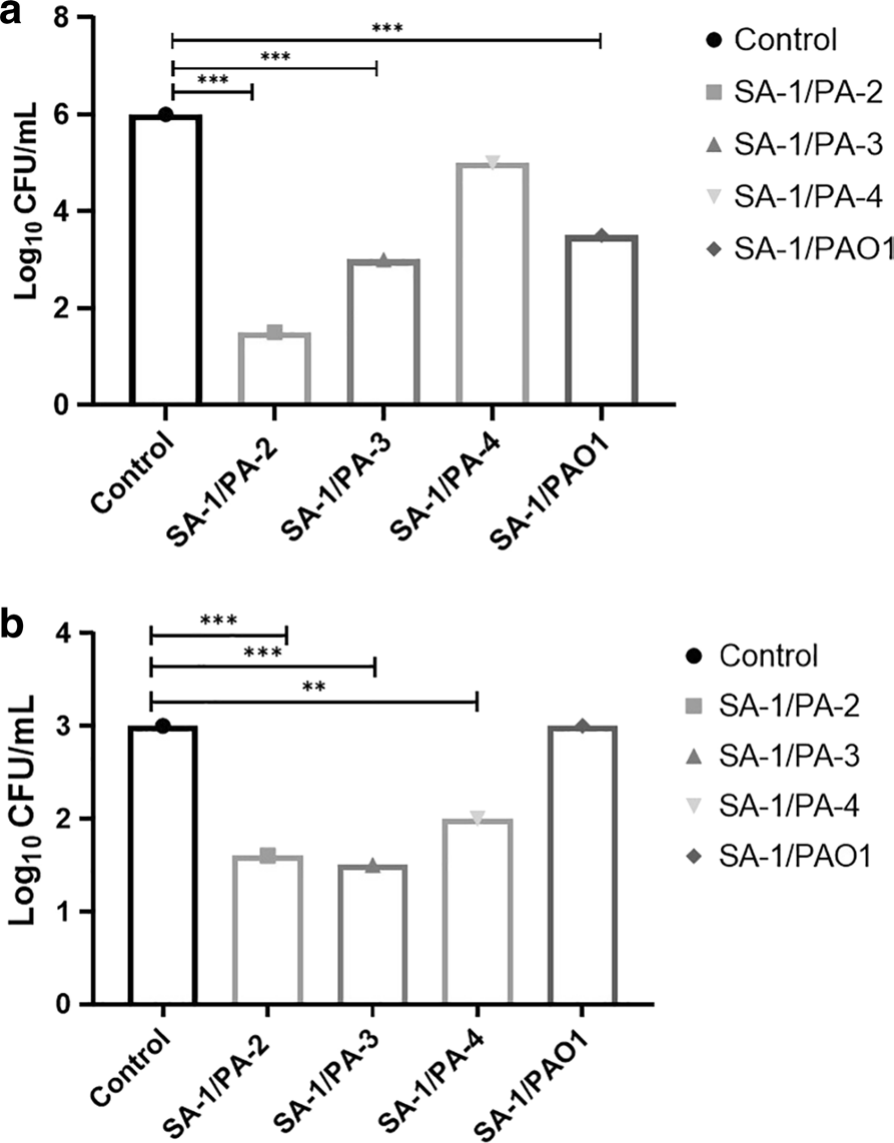
The planktonic (**a**) and biofilm (**b**) states of the co-culture of *P. aeruginosa—S. aureus* on the A549 cell line. The viability of *P. aeruginosa* was measured as log_10_ (CFU/well) in the co-culture with *S. aureus*. The error bars indicate the standard errors of the means in a representative triplicate time. '. *: p < 0.05, **: p < 0.001, ***: p < 0.0001

As demonstrated in Fig. 1a, the viability of *P. aeruginosa* decreased in the planktonic state of all strains. Comparing to the control group, the colony counts of PA-2, PA-3, and PAO1 decreased significantly during the co-culture with SA-1 (P-value $\le 0.001).$ In contrast to other strains, the colony count of PA-4 strain after co-culture indicated a slight reduction. Similar to the planktonic condition, the viability decreased in the biofilm state. A dramatic inhibitory effect of *S.aureus* on *P.aeruginosa* strains was observed in PA-2 and PA-3 comparing to PA-4 (Fig. 1b). According to the Student's t-test and χ2 test analysis (Fig. 1), the viability of *P. aeruginosa* strains decreased significantly during the biofilm state of the co-culture containing SA-1/PA-2, SA-1/PA-3, and SA-1/PA-4 ((P:0.0004), (P:0.0006), and (P:0.009), respectively). Conversely, there was no significant difference between the viability of the SA-1/PA-4 co-culture in the planktonic state (P: 0.59) based on the Student's t-test. All the other co-culture combinations had a strong and impressive effect on viability.

### Antibiotic resistance decreased in the recovered *P. aeruginosa* strains

**Fig. 2:**
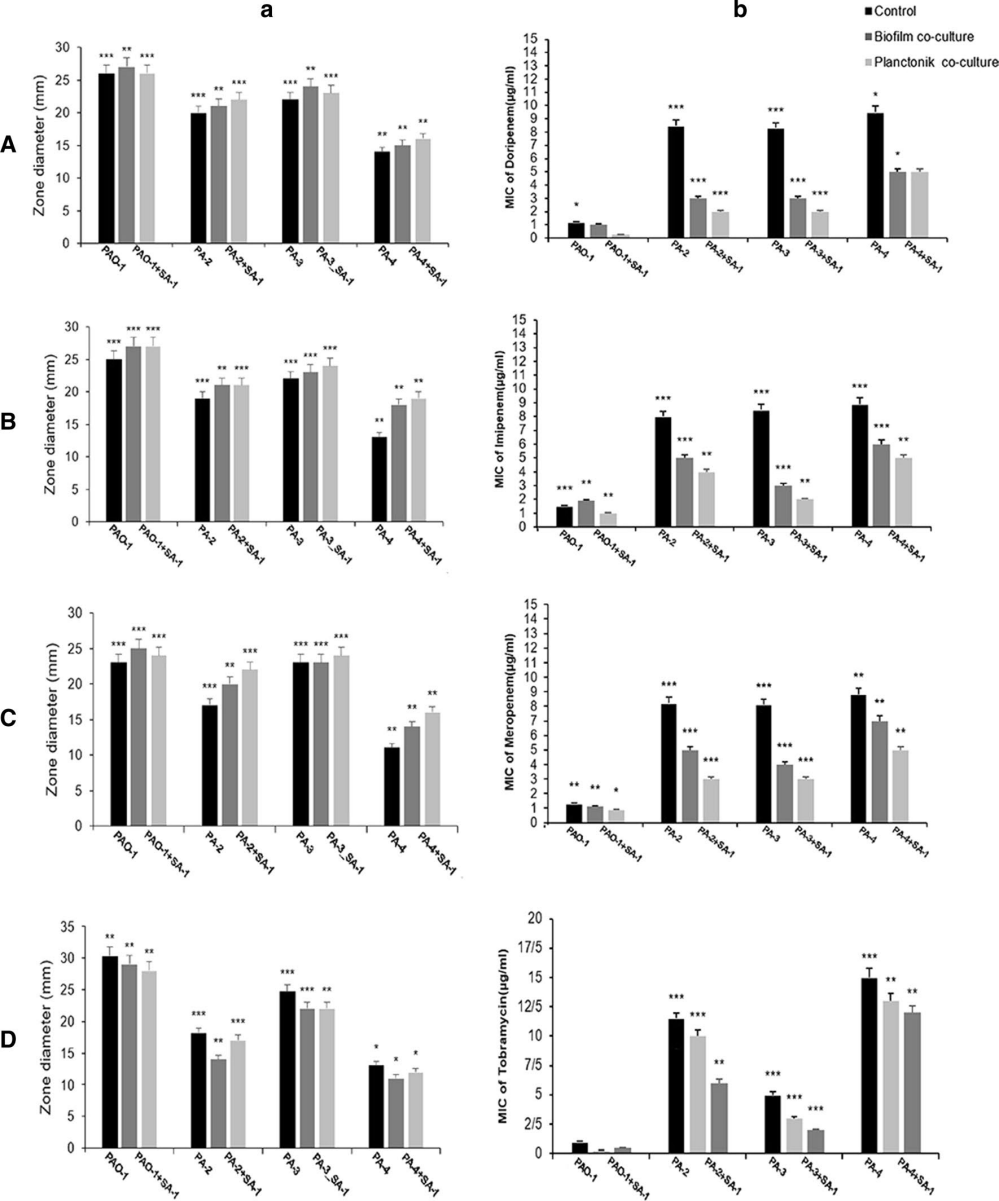
The effect of *S. aureus* on the antibiotic resistance of *P.aeruginosa* strains. The inhibition zone (**a**) and the MIC (**b**) of antibiotics is demonstrated. The MICs of doripenem (**A**), imipenem (**B**), meropenem (**C**), and tobramycin (**D**) on the *P. aeruginosa* strains. '*: p < 0.05, **: p < 0.001, ***: p < 0.0001. No stars: the non-statistical correlations

Figure 2 illustrates the antibiotic susceptibility patterns of the recovered strains of *P. aeruginosa*. The inhibition zone of antibiotics increased for carbapenems. However, the antibiotic susceptibility pattern of tobramycin was slightly different among the four strains. The findings of the MIC test indicated a significant decrease in the strains recovered from co-culture. The MIC of meropenem, imipenem, and doripenem showed a significant reduction for all recovered strains, except for imipenem's MIC in the biofilm state of PAO1 comparing to the control (Fig. 2a–c).

Moreover, antibiotic susceptibility increased more efficiently in the planktonic form comparing to the biofilm one. In other words, the MICs reduced from 8–9 μg/mL to 2–5 μg/mL in the planktonic state. While in the biofilm condition, the MIC of carbapenems was between 3 and 7 μg/mL.

The increase of carbapenems susceptibility was not similar among four strains of *P.aeruginosa*. Based on the Fig. 2, the MIC of carbapenems decreases from 8–9 to 2–3 μg/mL in PA-2 and PA-3 in comparison to PA-4 (reduced from 9 to 5 μg/mL). Inconsistent with the MIC results, carbapenems' inhibition zone increased more significantly for pathogenic strains than the MDR strain. According to the Student's t-test and χ2 test analysis (Fig. 2), carbapenemase mediating resistance reduced considerably in combinations containing SA-1/PA-2 and SA-1/PA-3 (P-value ≤ 0.001). In contrast to the SA-1/PA-4 combination, the co-culture of SA-1 with PA-2 and PA-3 led to a notable decline in resistance to carbapenems. Based on the two-way ANOVA test, there was a statistically significant relationship between the SA-1 strain and reduction of resistance to carbapenems in the co-culture. Inconsistency with the recovered strains' antibiotic resistance pattern, the *KPC* gene expression level remarkably decreased in PA-2 and PA-3 (P-value ≤ 0.001). Also, a slight decline of *KPC* expression level was observed in the PA-4 strain (P-value ≤ 0.001).

**Fig. 3:**
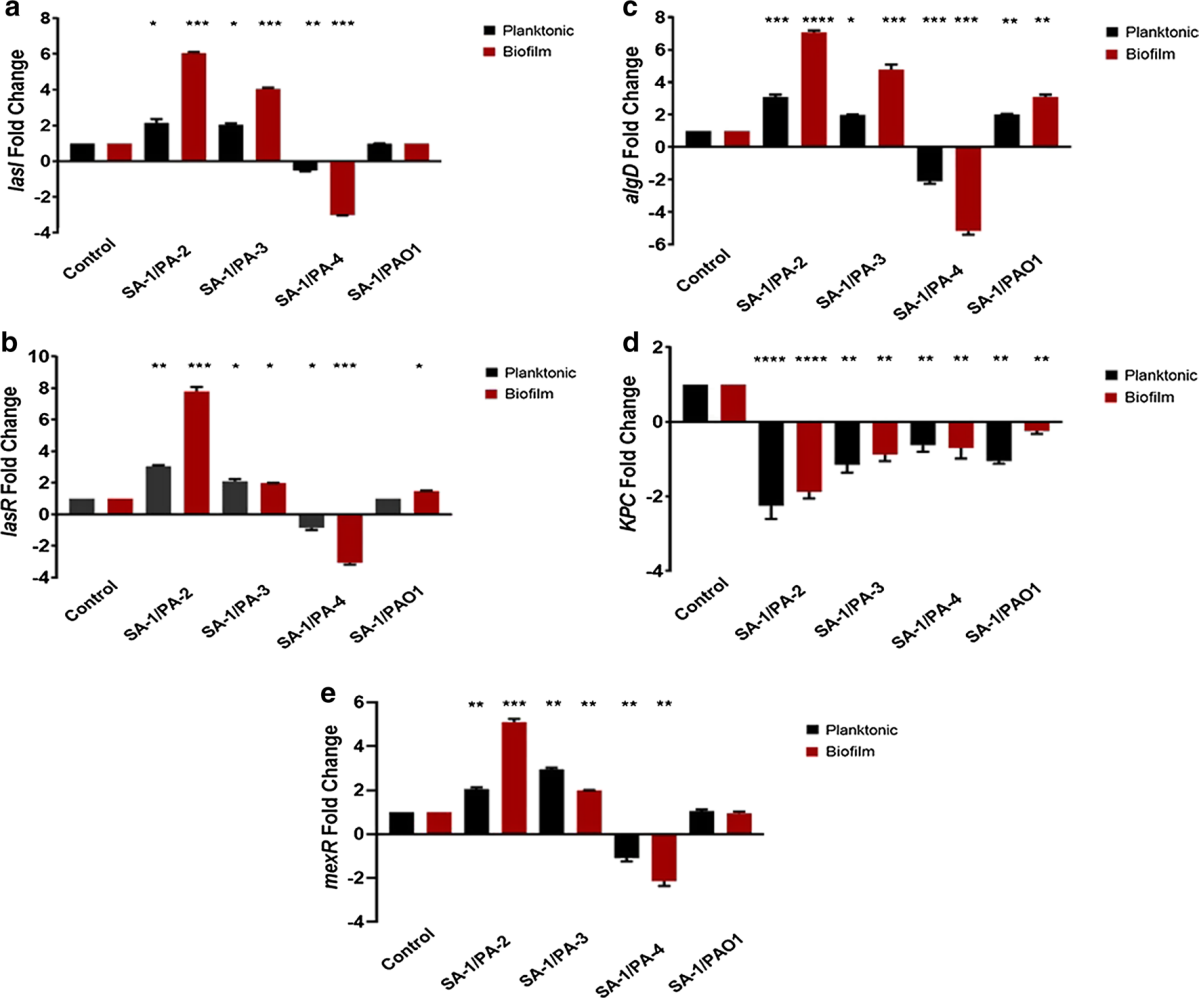
The expressions of *lasI* (**a**), *lasR* (**b**), *algD* (**c**), *KPC* (**d**), and *mexR* (**e**), in *P. aeruginosa*. The fold changes of the biofilm form in the expressions of *lasR*, *lasI*, *algD*, *mexR*, and *KPC* for the infected A549 cells as determined by RT-qPCR. The error bars indicate the standard errors of the means from a representative triplicate time. '*: *p* < *0.05*, **: *p* < *0.001*, ***: *p* < *0.0001*. No stars: the non-statistical correlations

According to panel D of Fig. 2, the inhibition zone and MIC of tobramycin showed a contradictory pattern in four strains of *P.aeruginosa*. While the MIC decreased from 5 to 2–3 µg/mL for PA-3 and PAO1 recovered from co-culture with SA-1, tobramycin resistance increased for PA-2 and PA-4 strains either in planktonic and biofilm states. Regarding Fig. 3e, the expression level of *mexR* as a negative regulator of efflux pumps decreased considerably in PA-2 and PA-4 during the co-culture (P-value ≤ 0.001). In contrast, a significant rise was observed in SA-1/PA-3 and SA-1/PAO1 combinations (P-value ≤ 0.001).

The two-way ANOVA test and Tukey's data analysis indicated a unique relationship between co-culture and antibiotic susceptibility (P-value ≤ 0.001). Moreover, the Student's t-test and two-way ANOVA test confirmed the statistically significant association between the antibiogram pattern and the co-culture, the gene expression level in the wild type, and the recovered strains.

### Virulence of *P. aeruginosa* was influenced by S. aureus in a strain-dependent manner

**Fig. 4:**
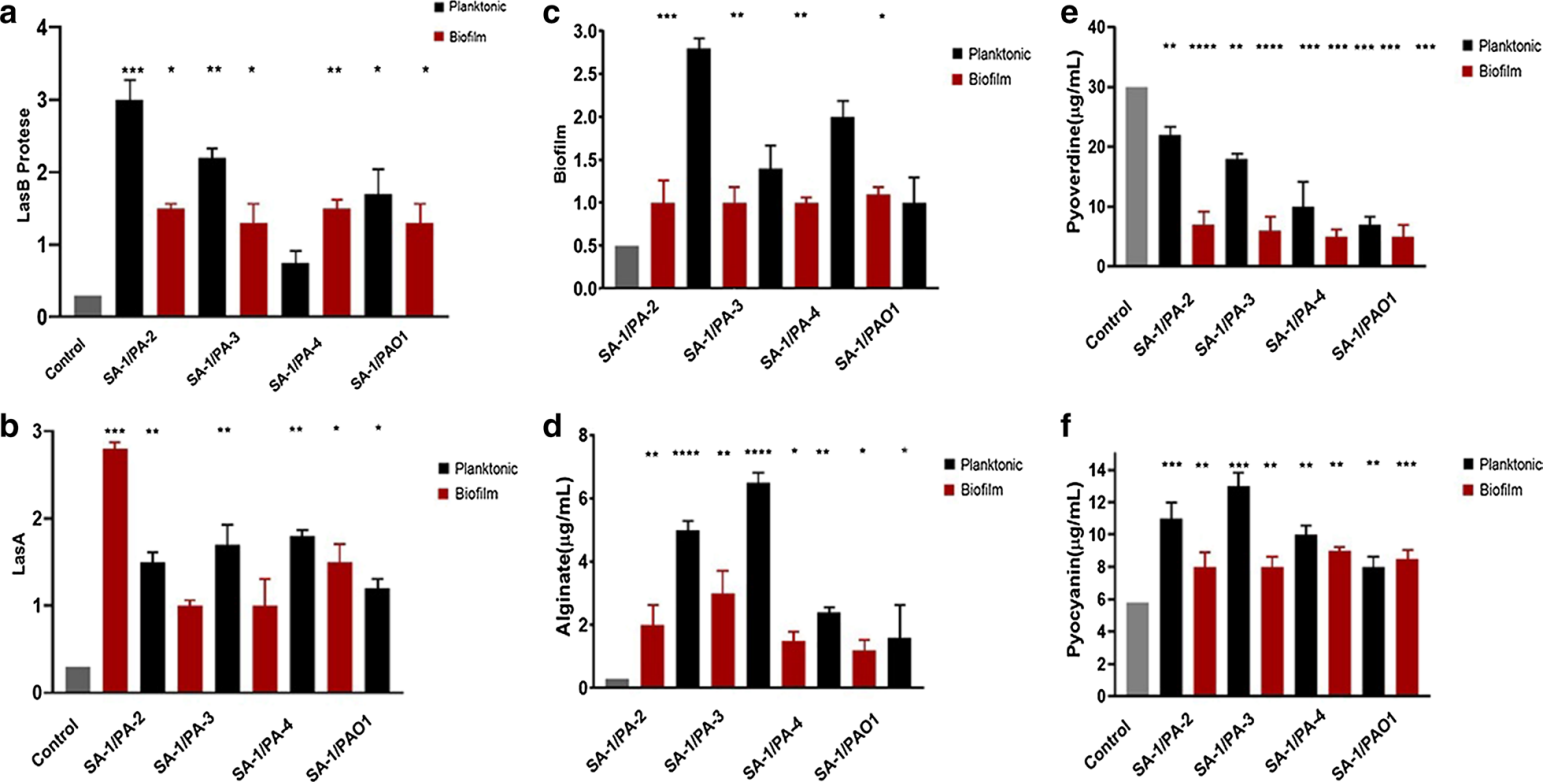
The changes in virulence factor production in *P.aeruginosa* strains. LasB protease (**a**), LasA protease (**b**), biofilm formation of *P. aeruginosa* (**c**), alginate production (**d**), pyoverdine (**e**), and pyocyanin (**f**) were investigated. ' *: p < 0.05, **: p < 0.001, ***: p < 0.0001. No stars: the non-statistical correlations

As demonstrated in Fig. 3a and b, the Las system up-regulated in all combinations except for SA-1/PA-4 in biofilm and planktonic co-culture states. Moreover, the virulence factors controlled by the Las system, including Las B, Las A, and biofilm formation, increased significantly (Fig. 4a–c). Two-way ANOVA and Tukey's tests indicated the significant effect of SA-1 on the *lasI/R* expression level and virulence factor production in PA-2 and PA-3, particularly in the biofilm state co-culture (P-value ≤ 0.001). Furthermore, the more significant modulatory effect of SA-1 on the Las system and virulence factor production was observed in the biofilm state comparing to the planktonic condition (P-value $\le 0.0001 vs. \text{P-value} \le 0.01)$. Conversely, a reduction in the activity of *lasI/R* and virulence factors was observed in PA-4, and no statistically significant, slight change in PAO1 strain (Figs. 3a, b, and 4). Also, pyocyanin production increased in PA-2 and PA-3, but pyoverdine concentration in PA-3 reduced compared to wild type. The two-way ANOVA analysis revealed a significant strain-dependent effect of *S. aureus* on the virulence of *P. aeruginosa* (P-value ≤ 0.001) (Fig. 4e, f).

During the co-culture of *P. aeruginosa* strains with SA-1, the alginate production multiplied notably, and the non-mucoid strains turned into the mucoid ones (P-value $\le 0.0001)$. Although the *algD* was up-regulated in the biofilm state, particularly in PA-2 and PA-3, it was down-regulated in PA-4 either in planktonic and biofilm conditions (Figs. 3c and 4d).

## Discussion

According to this study's findings, the viability of *P. aeruginosa* decreased in the planktonic state of all strains. However, a Significant reduction of PA-2, PA-3, and PAO1 were observed during the co-culture with SA-1 (P-value $\le 0.001).$ Unlike other strains, the colony count of PA-4 strain after co-culture indicated a slight decrease. Also, the *P. aeruginosa* recovered populations in the planktonic form were more than the biofilm state. A weaker effect of *S. aureus* on *P.aeruginosa* PA-2 and PA-3 strains was reported in comparison with *P.aeruginosa* PA-4. Nevertheless, the viability of *P. aeruginosa* strains decreased significantly during the biofilm state of the co-culture containing SA-1/PA-2, SA-1/PA-3, and SA-1/PA-4 ((P:0.0004), (P:0.0006), and (P:0.009), respectively). Based on these findings, the growth of *P.aeruginosa* was restrained by *S.aureus*, as it was confirmed in previous studies (Hotterbeekx et al. 2017; Alves et al. 2018), suggested that *S. aureus* had a more inhibitory effect on the pathogenic strains of *P. aeruginosa*. Frapwell et al. (2018) and Ali Mirani et al. (2018) demonstrated that in the biofilm condition, the viability of *P. aeruginosa* was reduced after three days which was the most important reason for the type of metabolic pathway and the occurrence of genetic mutations in these bacteria. According to Orazi and O'Toole (2017), the interaction between *P. aeruginosa* and *S. aureus* in co-culture conditions alters the metabolic pathway of the former one and the metabolism shifts to fermentative growth and reduced antibiotic resistance. The most notable repressive effect of *S.aureus* was observed for the PA-2 strain. The carbapenem resistance decreased remarkably in this strain, and the Las system and Las mediated virulence factors increased notably.

Based on the current study and the findings of the MIC test, our results indicated a significant decrease in the strains recovered from co-culture, except for imipenem's MIC in the biofilm state of PAO1 comparing to the control.Moreover, antibiotic susceptibility increased more efficiently in the planktonic form comparing to the biofilm one. However, in PA-2 and PA-3 strains, the MIC of carbapenems decreases from 8 to 9 to 2–3 μg/mL. Also, in those strains, carbapenems' inhibition zone increased more significantly than the MDR strain. In other words, carbapenemase mediating resistance reduced considerably in combinations containing SA-1/PA-2 and SA-1/PA-3 (P-value $\le$ 0.001). Unlike the other strains, the PA-2 strain demonstrated an increase in tobramycin resistance. Increased resistance to tobramycin in the biofilm-producing strains of *P. aeruginosa* in co-culture with *S. aureus* was reported in the study of Beaudoin et al. (2017). Furthermore, the inhibitory effect of SA-1 on *P. aeruginosa* strains was more significant in the planktonic state comparing to the biofilm condition, and as it was predicted, this effect was observed moderately less in MDR strain in both conditions of co-culture in consistence to the studies of Chan et al. (2018) and DeLeon et al. (2014). Yang et al*.* (2011) also suggested that biofilm formation may be a beneficial survival characteristic in the co-culture.

Our results demonstrated resistance to carbapenems decreased in the PA-2 and PA-3 strains so that the MIC changed from 8 to 4 μg/mL. Moody (2014), Frapwell et al. (2018), and Tognon et al. (2019) showed that *S. aureus* reduced the antibiotic resistance of *P. aeruginosa* in co-culture conditions. Thus, there was a significant correlation between SA-1 and the viability of *P. aeruginosa* strains (P-value $\le$ 0.0001, P-value $\le$ 0.0002, and P-value $\le$ 0.0001 for PA-2, PA-3, and PA-4, respectively). Hence, based on the findings of this study, *S. aureus* in the co-culture condition had a remarkable effect on viability and resistance of *P. aeruginosa*, as it was reported in the study of Frapwell et al. (2018). However, our results conformed a unique relationship between co-culture and antibiotic susceptibility (P-value $\le 0.001).$

Based on the present study, *lasI/R* and its dependent virulence factors up-regulated in all combinations except for SA-1/PA-4 in biofilm and planktonic co-culture states. Thus, significant effect of SA-1 on the *lasI/R* expression level and virulence factor production in PA-2 and PA-3, particularly in the biofilm state co-culture (P-value $\le 0.001)$, was observed. Besides, SA-1 strain had a more inhibitory effect on the Las system and virulence factor production in the biofilm state compared to the planktonic state (P-value $\le 0.0001 \text{vs. P-value} \le 0.01)$. The studies by Kim et al. (2015), Radlinski et al. (2017), and Yang et al. (2011) confirmed that the virulence of *P. aeruginosa* altered by interaction with *S. aureus*. However, a reduction in the activity of *lasI/R* and virulence factors was observed in PA-4, and no statistically significant, slight change in PAO1 strain. Woods et al. (2019) observed that the expression levels of the *lasI/R* of *P. aeruginosa* in the co-culture with *S. aureus* increased. Furtehr, *lasI* and *algD* genes expression levels in biofilm- and toxin-producing strains were more pronounced than the carbapenem-resistant strain.; however, no increase was observed in the PA-4. Similar results were reported by Limoli et al. (2016) and Tognon et al. (2019). The KPC activity decreased in all *P. aeruginosa* strains, and the lowest activity of this gene was detected in the PA-4 strain. DeLeon et al. (2014) used the term 'mutual benefit' for *P. aeruginosa* and *S. aureus* in cell culture conditions.

In the current study, pyocyanin production increased in PA-2 and PA-3. Howeer, pyoverdine concentration in PA-3 reduced compared to wild type. Oue analysis revealed a significant strain-dependent effect of *S. aureus* on the virulence of *P. aeruginosa* (P-value $\le 0.0001).$ The studies by Kim et al. (2015), Radlinski et al. (2017), and Yang et al. (2011) confirmed that the virulence of *P. aeruginosa* altered by interaction with *S. aureus*. Furthermore, the production of pyoverdine was reduced in all strains of *P. aeruginosa* except PA-4. Abisado et al. (2018) and Yang et al. (2011) demonstrated that *S. aureus* had a more significant inhibitory effect on the virulence and biofilm production of *P. aeruginosa* in an in vivo co-culture. Koley et al. (2011) showed that pyocyanin created a redox potential gradient in the biofilm called electro line, which increased iron availability.

This change provides the basis for the increased pathogenicity of *P. aeruginosa* in fermentative conditions. Another important issue addressed in the current study, also confirmed by the studies of Armbruster et al. (2016) and Alves et al. (2018) is the increased production of pyoverdine and pyocyanin after the co-culture in PA-3. According to the study of Hotterbeekx et al. (2017), there was a significant relationship between the increased pathogenicity of *P. aeruginosa* and the effects of *S. aureus*. They found that in polymicrobial infections, *S. aureus* increased the virulence factors of *P. aeruginosa*, which confirms our results.

A limitation of the current study is that many variables can affect the growth of the bacterium in the A549 cell line. Therefore, it is suggested that in future studies, the nutritional status of the cell be taken into account when evaluating QS-based gene expression. QS-based gene regulation models based on planktonic cells' studies must be modified to explain the behavior of biofilm gene expression since gene expression in biofilms is dynamic. Besides, determining the physiological differences between biofilm and planktonic cultures is critical for understanding *P. aeruginosa* infections (such as those found in the cystic fibrosis lung) or removing problematic biofilms from tissue infections.

In conclusion, our findings demonstrated that an initial foundation is needed to explain how factors other than cell density can control the expressions of quorum sensing-regulated genes and carbapenemase genes. Since biofilm formation, toxicity, and carbapenem resistance cause the up-or down-regulation of quorum sensing regulated genes (*lasR/lasI*), it is not inconceivable that globally regulated genes can be controlled by more than one factor. Even though this conclusion is novel, it is not surprising. Furthermore, in the co-culture in the A549 cell line, a significant relationship was observed among the viability of *P. aeruginosa*, the activity of pathogenic enzymes, incubation time, resistance to carbapenem, and the expression of virulence genes. Carbapenemase enzymes played a more critical role than pathogenic enzymes in maintaining bacterial growth. Hence, in respiratory infections, resistance to carbapenem antibiotics in *P. aeruginosa* can provide a basis for the development and spread of co-infection with *S. aureus*. Besides, the production of pathogenic enzymes and biofilms by *P. aeruginosa* changes the metabolic pathways of the bacteria and causes the emergence of pathogenic strains.

## Data Availability

The datasets used and/or analyzed during the current study are available from the corresponding author on reasonable request.

## Footnotes

1 KPC: *Klebsiella pneumoniae* carbapenemase.
